# Modulation of GABA by sodium butyrate ameliorates hypothalamic inflammation in experimental model of PCOS

**DOI:** 10.1186/s12868-023-00834-z

**Published:** 2023-11-23

**Authors:** Oony-Iye Eepho, Al-Amin M. Bashir, Adesola A. Oniyide, Ayodeji Aturamu, Olutunmise V. Owolabi, Isaac O. Ajadi, Adedamola A. Fafure, Mary B. Ajadi, Stephanie E. Areloegbe, Kehinde S. Olaniyi

**Affiliations:** 1 Cardio/Endo-metabolic and Microbiome Research Unit, Department of Physiology, College of Medicine and Health Sciences, Afe Babalola University, P.M.B. 5454, Ado-Ekiti, 360101 Nigeria; 2 Department of Biochemistry, College of Medicine and Health Sciences, Afe Babalola University, Ado-Ekiti, 360101 Nigeria; 3 Department of Physiology, Faculty of Basic Medical Sciences, Ladoke Akintola University of Technology, Ogbomosho, Nigeria; 4 Department of Anatomy, College of Medicine and Health Sciences, Afe Babalola University, Ado-Ekiti, 360101 Nigeria; 5 Department of Chemical Pathology, College of Health Sciences, Ladoke Akintola University of Technology, Ogbomosho, Nigeria

**Keywords:** Butyrate, Inflammation, Hypothalamus, PCOS, GABA

## Abstract

Polycystic ovarian syndrome (PCOS) is a known endocrine disorder that has affected many women of childbearing age, and is accompanied by various neurodegenerative conditions. Hence, this study investigates the impact of butyrate in reversing hypothalamic-related disorder, possibly through γ aminobutyric acid (GABA) in a rat model of PCOS. Eight-week-old female Wistar rats were allotted into four groups (n = 5), which include control, butyrate, letrozole, and letrozole + butyrate groups. PCOS was induced by administering 1 mg/kg of letrozole (oral gavage) for 21 days. After confirmation of PCOS, 200 mg/kg of butyrate (oral gavage) was administered for 6 weeks. Rats with PCOS were characterized by elevated levels of plasma insulin and testosterone. Increases in plasma and hypothalamic triglyceride levels, inflammatory biomarker (SDF-1), apoptotic marker (caspase-6), and decreased plasma GnRH were observed. Additionally, a decrease in hypothalamic GABA was revealed. Nevertheless, the administration of butyrate attenuated these alterations. The present study suggests that butyrate ameliorates hypothalamic inflammation in an experimental model of PCOS, a beneficial effect that is accompanied by enhanced GABA production.

## Introduction

Polycystic ovarian syndrome (PCOS) stands as the most common hormonal disorder in females of reproductive age [1]. A multifactorial endocrine disorder with hyperandrogenism and insulin resistance (IR) as the core etiologic and primary attributes [2, 3], characterized by irregular cycles, ovulatory dysfunction, hyperandrogenism and polycystic ovarian morphology (PCOM) [4], This reproductive endocrine disorder can be caused by genetic, epigenetic and environmental factors or lifestyle [4]. This multifactorial disorder still poses a serious issue to women of childbearing age. At an estimate between 4% and 20%, the global prevalence of PCOS remains to be a major concern worldwide [5]. Around 5–15% of women in the childbearing age group suffer from hormonal imbalances that lead to menstrual irregularities, infertility, and other health disorders that include cardiovascular complications, endometrial cancer, and type 2 diabetes mellitus (T2DM) [6]. The World Health Organization (WHO) data suggests that over 166 million women are affected by PCOS on a global basis [7]. Despite PCOS being the most common endocrine health problem of women of reproductive age, its management remains a significant challenge to the medical profession [8].

Through a feedback loop called the hypothalamic-pituitary-ovarian (HPO) axis, the hypothalamus interacts with the pituitary gland and the ovaries [9]. A disruption of this axis in women with PCOS leads to abnormal levels of hormones such as the luteinizing hormone (LH), follicle stimulating hormone (FSH), and androgens, resulting in irregular periods, infertility, polycystic ovaries, hirsutism, acne and metabolic problems, with obesity and insulin resistance (IR) among the main factors influencing the clinical manifestations of PCOS. These deleterious changes can be caused by genetic factor or lifestyle [10]. Androgen synthesis and secretion are enhanced directly or indirectly by impaired insulin activity; increased free fatty acids resulting from stimulation of breakdown of adipose tissue (AT) due to elevated androgen levels augments IR [11]. Insulin also affects the central nervous system (CNS) by actions in peripheral glucose metabolism and energy homeostasis [12]. Therefore, alterations to insulin metabolism in the CNS may pose detrimental effects on cognitive activity and hypothalamic function [13]. Additionally, chronic inflammation as a result of obesity and/or other metabolic-related disorders remains a serious disturbance to the PCOS patient [14]. Hypothalamic inflammation exhibits characteristics of chronic low-grade inflammation, notably at the molecular level [15, 16]. Recently, advanced glycosylation end products (AGEs) and their receptors implicated in the inflammation and oxidative stress cascades have also been found to be overexpressed in PCOS women [17]. Nonetheless, taking into account the crucial role of HPO axis in the pathogenesis of PCOS, therapeutic targeting the HPO axis can be effective [18], the exact cause of hypothalamic dysfunction remains unclear in the pathogenesis of PCOS.

The disruption of central nervous system is a major component of neurodegeneration [19]. In understanding the neuroendocrine pathology of PCOS, the hypothalamus circuits that have received the most attention include the arcuate kisspeptin neurons recently identified as the GnRH neuron pulse generator [20, 21] and GABAergic neurons [21], there are various upstream neural and endocrine factors that contribute to both the timing and magnitude of GnRH secretion, with the stimulation of GnRH neurons by GABA occurring through the GABA receptor [20]. Neuroinflammation is initiated by microglia, which are the resident immune cells of the CNS. As an effector, microglia affect neuronal network development and progression of neurodegenerative disorders. GABA release from astrocytes appears to be a mechanism to regulate and suppress pro-inflammatory signaling in microglia [21, 22].

Short chain fatty acids are the product of fiber fermentation by the gut microbiota. They can be locally metabolized, or absorbed and transported for further metabolism to the liver and they include mainly acetate, propionate and butyrate [23]. The intake of high fiber has been linked to the improvement of metabolic syndrome by increasing energy expenditure and improving insulin sensitivity. Accumulating literatures describe butyrate as a link between diet and metabolic health of the host [24]. However, the neuroprotective effect of butyrate particularly in a model of PCOS is unclear. Therefore, the present study sought to elucidate the impact of SCFA, butyrate on hypothalamic inflammation in a rat model of PCOS. In addition, the study also attempted to investigate the possible involvement of GABA in PCOS.

## Materials and methods

### Ethical approval

The current study was conducted in conformity with the National Institutes of Health Guide for the Care and Use of Laboratory Animals, and approval of the protocol was obtained from Independent Ethical Review Board of Afe Babalola University (Nigeria).

### Experimental animals and grouping

Female Wistar rats of eight-weeks old were procured from the animal house of College of Medicine and Health Sciences, Afe Babalola University. The rats were allowed free access to tap water and standard rat chow. The rats utilized for the present study were observed with at least three consecutive regular estrous cycles with the same estrous stage, which was determined through vaginal smear. Animals were acclimatized for a week, the rats were then randomly allotted into four groups with n = 5 per group, designated as; control (CTR), sodium butyrate (SOB), letrozole (LTZ) and LTZ + SOB groups. Subject to standard environmental conditions, rats were maintained in a colony with (22–26 ^0^ C of temperature), (50–60% of relative humidity), and a 12-hour dark/light cycle.

### Induction and confirmation of PCOS

Polycystic ovarian syndrome was induced in experimental rats by administration of 1 mg/kg body weight of letrozole (oral gavage; Sigma-Aldrich, St. Louis, MI.) for a duration of 21 days as documented previously [25, 26]. The manifestation of PCOS was confirmed using Rotterdam criteria [27].

### Treatment

Control group received vehicle (distilled water, oral gavage), SOB group received sodium butyrate (200 mg/kg, oral gavage, Sigma-Aldrich, St. Louis, MI.), LTZ group received distilled water and LTZ + SOB group received sodium butyrate, with the administration lasting for six weeks [28].

### Collection of samples

At the conclusion of treatment, the rats were fasted overnight and anesthetized by injecting (*ip*) 50 mg/kg body weight of sodium pentobarbital. Thereafter, blood sample was collected via cardiac puncture into heparinized tube and centrifuged at 704 *g* for 5 min at room temperature. Plasma was stored at -80 °C for two weeks before the biochemical assays. After perfusion with normal saline to flush blood out of the vascular system, the hypothalamus was removed, homogenized in 1 ml of phosphate buffer solution. Thereafter, centrifuged at 8000 *g* for 10 min at 4 °C. The supernatant fluid was collected and stored at -80 °C for two weeks before the biochemical analysis.

### Biochemical analysis

#### Endocrine profile

Plasma concentration of insulin, testosterone and GnRH were determined using rat ELISA kit purchased from Calbiotech Inc. (Cordell Ct., El Cajon, CA 92,020, USA) in compliance with manufacturer’s guidelines.

#### Determination of lipid profile

The levels of triglyceride (TG) were determined in the plasma and hypothalamic tissue by standard colorimetric methods using assay kits obtained from Fortress Diagnostics Ltd. (Antrium, UK).

#### Assessment of pro-inflammatory and apoptotic markers

The concentration of stromal cell-derived factor-1 (SDF-1) was determined in the supernatant of the hypothalamic tissue homogenate using quantitative standard sandwich ELISA technique with a monoclonal antibody specific for these parameters obtained from Elabscience Biotechnology Inc. (Wuhan, Hubei, P.R.C., China), and cat. number E-EL-R3027, in adherence to the procedure provided by the manufacturer. The concentration of Caspace-6 was determined in the supernatant of the hypothalamic tissue homogenate using rat ELISA kit, obtained from ELK Biotechnology Co. Ltd. (1312 17th Street #692 Denver, CO 80,202 USA) with cat. Number ELK8812.

#### Determination of GABA levels

The level of GABA was determined from the supernatant of the hypothalamic tissue homogenate by standardized enzymatic colorimetric method employing the use of an assay kit obtained from ELK Biotechnology Co. Ltd. (1312 17th Street #692 Denver, CO 80,202 USA) with cat number ELK1513.

### Statistical analysis

Statistical analysis was performed with GraphPad Prism software version 9, and all data were expressed as means ± SD. One-way ANOVA was used to compare the mean values of variables among the groups. Bonferroni’s test was used for *post hoc* analysis and statistical significant difference was considered at p less than 0.05.

## Results

### Effects of sodium butyrate on hormonal profile in letrozole-induced PCOS

**Fig. 1:**
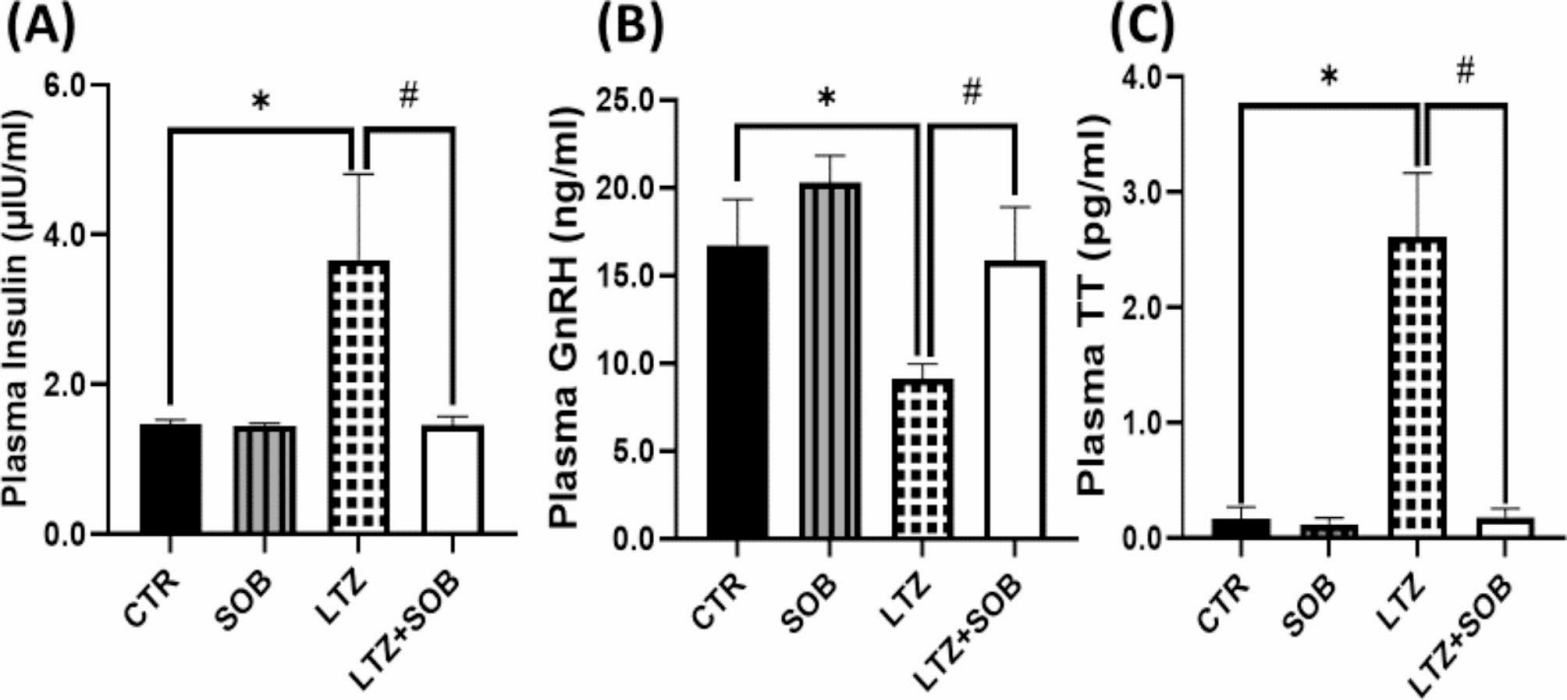
Effect of sodium butyrate on plasma insulin (**A**) plasma GnRH (**B**) and Plasma TT(**C**). Data are expressed as mean ± S.D. *n* = 5. Data were analyzed by one-way ANOVA followed by Bonferroni *post hoc* test. (*P < 0.05 vs. CTR; ^#^*P* < 0.05 vs. LTZ). Control (CTR); Sodium Butyrate (SOB); Letrozole (LTZ); Gonadotropin hormone (GnRH), Total testosterone (TT).

In plasma insulin and testosterone, a significant increase (*p* < 0.05) in LTZ group was observed compared with CTR. While a reduction (*p* < 0.05) in LTZ + SOB group was reported in comparison with LTZ group upon administration of sodium butyrate. Additionally, rats showed significantly lowered (*p* < 0.05) levels of plasma GnRH in LTZ group when compared with CTR group. Nonetheless, a significant increase (*p* < 0.05) in the LTZ + SOB group when compared with LTZ group was observed following administration of sodium butyrate as shown in Fig. 1.

### Effects of sodium butyrate on plasma and hypothalamic TG in letrozole-induced PCOS

**Fig. 2:**
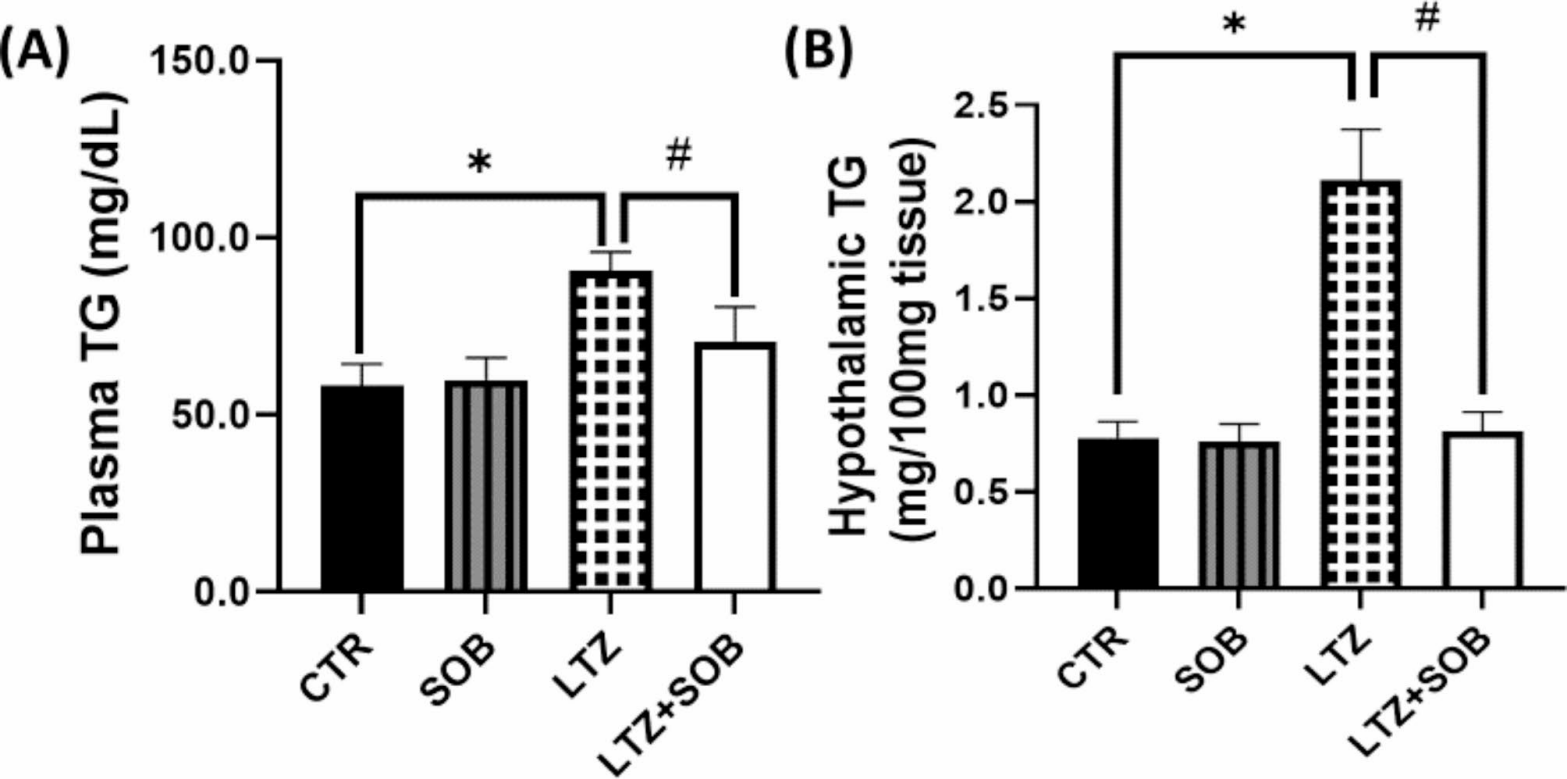
Effect of sodium butyrate on plasma TG (**A**) and hypothalamic TG (**B**). Data are expressed as mean ± S.D. *n* = 5. Data were analyzed by one-way ANOVA followed by Bonferroni *post hoc* test. (*P < 0.05 vs. CTR; ^#^*P* < 0.05 vs. LTZ). Control (CTR); Sodium Butyrate (SOB); Letrozole (LTZ); Triglyceride (TG).

Animals with PCOS showed a significant increase (*p* < 0.05) in plasma TG level of LTZ group when compared with CTR group. However, in the LTZ + SOB group, there was a reduction (*p* < 0.05) in plasma TG compared with LTZ group. Moreover, hypothalamus TG level was increased (*p* < 0.05) in LTZ group compared to the CTR group. Additionally, significant decrease (*p* < 0.05) in hypothalamic TG level in LTZ + SOB group was observed when compared with LTZ group following sodium butyrate administration as illustrated in Fig. 2.

### Effects of sodium butyrate on inflammatory and apoptotic biomarkers in letrozole-induced PCOS

**Fig. 3:**
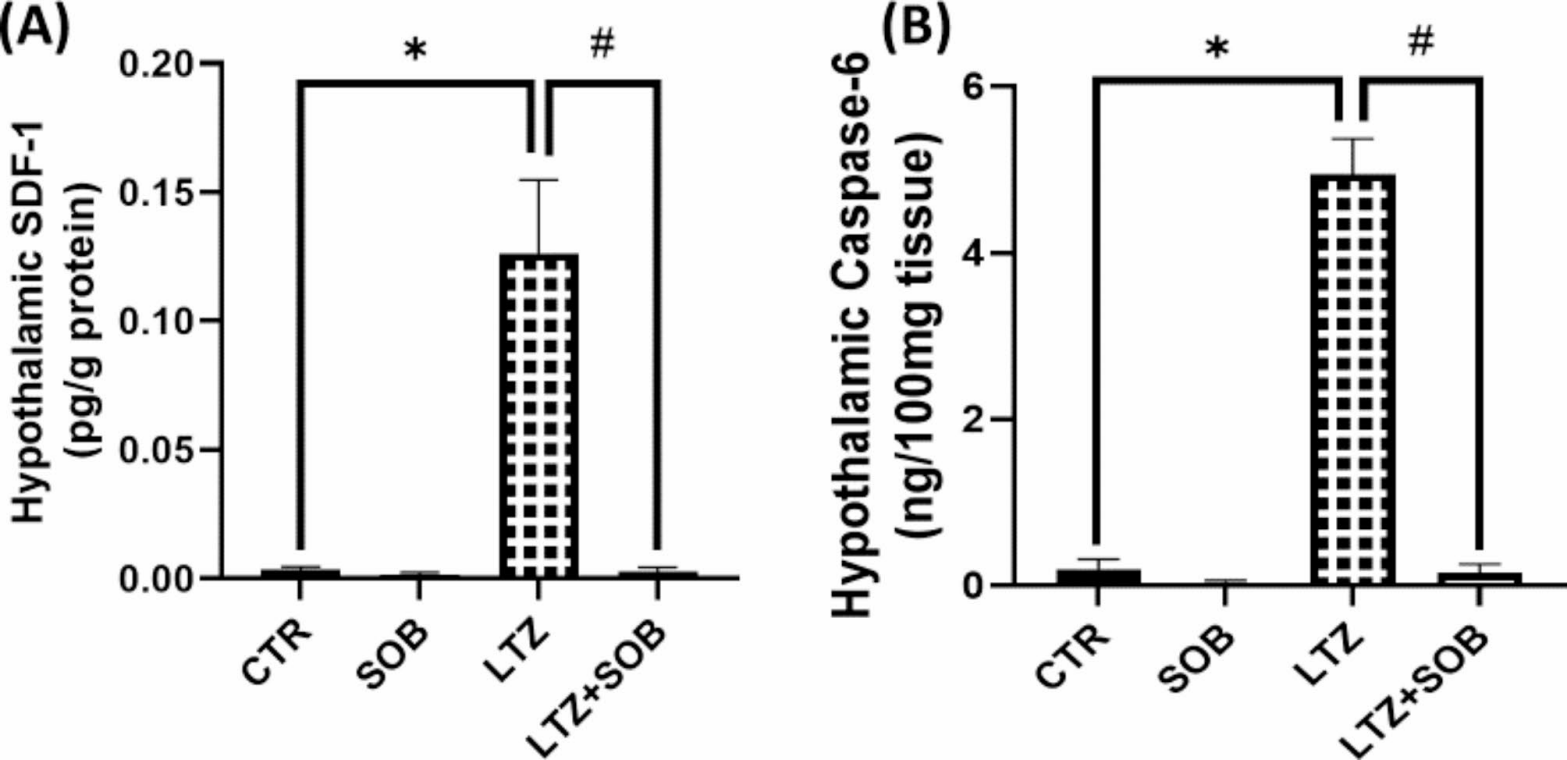
Effect of sodium butyrate on hypothalamic SDF-1 (**A**) and Caspase-6 (**B**). Data are expressed as mean ± S.D. *n* = 5. Data were analyzed by one-way ANOVA followed by Bonferroni *post hoc* test. (*P < 0.05 vs. CTR; ^#^*P* < 0.05 vs. LTZ). Control (CTR); Butyrate (SOB); Letrozole (LTZ); (Stroma cell-derived factor 1 (SDF-1).

PCOS-induced (LTZ) animals significantly increased (*p* < 0.05) hypothalamic SDF-1 level when compared to CTR group, which was reversed by sodium butyrate in LTZ + SOB group compared with LTZ group. The levels of hypothalamic caspase-6 were elevated (*p* < 0.05) in the LTZ group when compared to CTR group. Conversely, when administered sodium butyrate, a significant decrease (*p* < 0.05) was observed in LTZ + SOB group compared with LTZ group as shown in Fig. 3.

### Effects of sodium butyrate on hypothalamic GABA in letrozole-induced PCOS

**Fig. 4:**
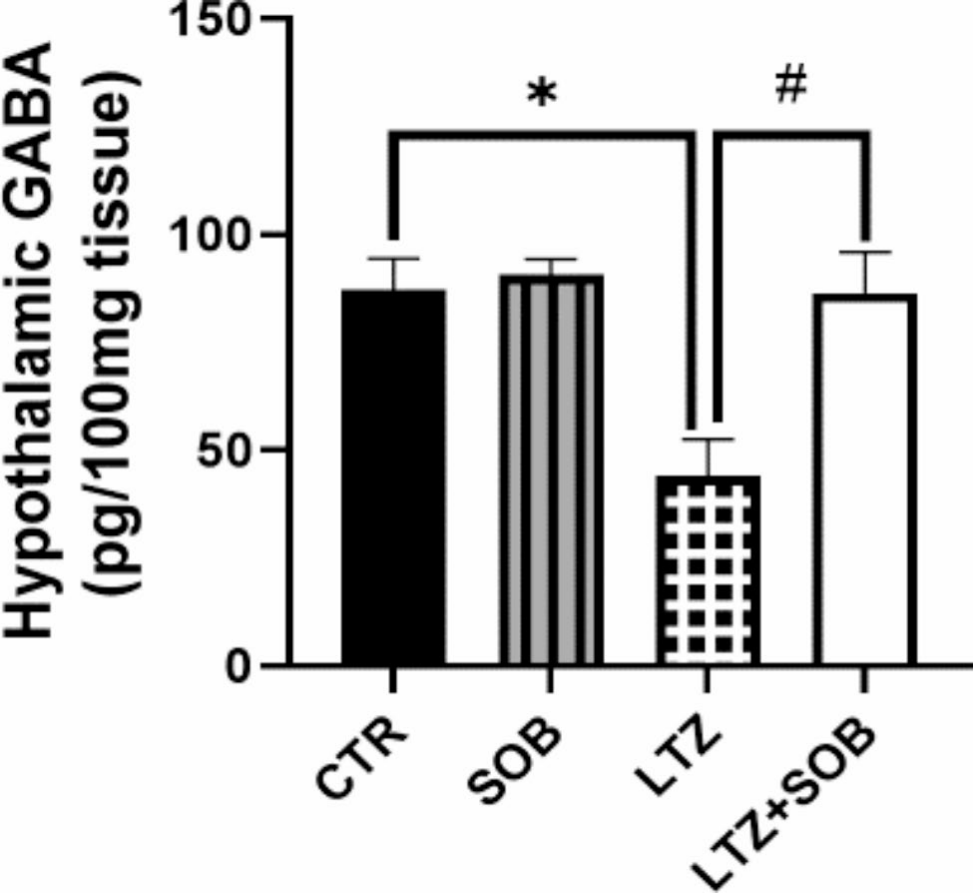
Effects of sodium butyrate on hypothalamic GABA in letrozole-induced PCOS. Data are expressed as mean ± S.D. *n* = 5. Data were analyzed by one-way ANOVA followed by Bonferroni *post hoc* test. (*P < 0.05 vs. CTR; ^#^*P* < 0.05 vs. LTZ). Control (CTR); Butyrate (SOB); Letrozole (LTZ); (Gamma aminobutyric acid (GABA).

There was a significant decrease (*p* < 0.05) in hypothalamic level of GABA in LTZ group compared with CTR. Furthermore, following administration of sodium butyrate, LTZ + SOB animals were shown with elevated (*p* < 0.05) levels of hypothalamic GABA when compared with LTZ group as shown in Fig. 4.

## Discussion

The key findings of the present study revealed that short chain fatty acid, butyrate attenuated hypothalamic inflammation in the multifactorial endocrine disorder, PCOS by improving GABA production. In addition, the results of this study also revealed that rats with PCOS were characterized with hyperinsulinemia, hyperandrogenemia and dyslipidemia. The levels of the chemokine, SDF-1, and the “executioner” caspase 6 in the hypothalamus were assessed. Importantly, the level of GABA was significantly reduced in LTZ group compared to control animals. Nonetheless, these alterations were reversed, highlighting the role of short chain fatty acid, sodium butyrate, in mitigating oxidative stress and hypothalamic inflammation in PCOS-induced animals.

Insulin regulates glucose homeostasis in the body. The present findings show a significant increase plasma insulin in animals with PCOS compared with control, confirming the metabolic defect in PCOS (16). Among other physiological functions, insulin may modulate neurotransmitter concentration through different mechanisms [29]. Insulin in addition plays a significant role as a long-term neuroprotectant, and its absence results in neurodegeneration [30]. This supports our data reporting a decreased level of GABA in PCOS-induced rats. However, administration of sodium butyrate significantly attenuated metabolic disruption in LTZ + SOB group with corresponding increase in hypothalamic level of GABA. At the cellular level, insulin resistance may be understood as the neuroplastic impairment or the neurotransmitter release in neurons. Looking at a multitude of central nervous system dysfunctions, chemokines are considered vital mediators and regulators of central inflammatory response [31, 32]. Furthermore, hypothalamic inflammation characterized in our findings with lipotoxicity (excess TG) and elevated SDF-1 lowers the integrity of hypothalamus, contributing to hypothalamic apoptosis (elevated caspase-6). Insulin resistance, hyperandrogenism and chronic low-grade inflammation may act together in a vicious cycle in the pathophysiology of PCOS [33].

There was a significant reduction in GnRH and elevated testosterone levels in PCOS animals. Although several factors are involved, resistance to the insulin and heightened androgen levels are considered the critical factors in PCOS pathogenesis, this is also associated with impaired steroid hormone negative feedback, driving excess androgen production in ovarian theca cells [34]. The surge in androgen secretion is associated with malfunctioning of islets of Langerhans, thereby compromising the pancreatic metabolic functions and causing hyperinsulinemia [35] as validated in the present study. A number of studies have demonstrated that many neurotransmitter and neuropeptide receptors are expressed in GnRH neurons, and they directly regulated the release of GnRH, influencing the action of LH and FSH on the ovarian tissue that aggravates androgen level.

Hyperandrogenemia-induced insulin resistance causes plasma and hypothalamic triglyceride increase in PCOS animals, resulting in hypothalamic lipotoxicity, which is characterized by excess lipid deposition in the hypothalamus of PCOS animals. Dyslipidemia is also a serious metabolic issue in PCOS, and as validated in this model, most patients with PCOS present dyslipidemia and insulin resistance with or without obesity [36, 37]. However, sodium butyrate reduced plasma and hypothalamic lipid deposition in LTZ + SOB group compared with LTZ group, indicating the antilipidemic effect of butyrate as previously documented [28].

Increased levels of pro-inflammatory markers have been reported in both hypothalamus and plasma of patients with neurodegenerative disorders. The release of inflammatory markers is further associated with long-term metabolic complications and high cardiovascular risk [3, 11]. Notably, this study revealed elevated levels of hypothalamic SDF-1 and caspase-6 in PCOS, reflecting the expression of inflammation and progression of hypothalamic apoptosis in PCOS animals. The protease apoptotic marker, caspase-6, is responsible for mediating apoptotic cell death. Increase in the level of chemokine, SDF-1, was associated with inflammation. These neuropathological markers differ in their etiologies but share common pathogenic manifestations such as neuroinflammation [38] and oxidative stress [39]. Also, chronic inflammation potentiates resistance to insulin and insulin-like growth factor-1 (IGF-1) in the hypothalamus, as presented in Alzheimer’s and Parkinson’s disease [30]. In addition, hypothalamic inflammation results in deregulation of peripheral insulin action [40], and a reduction in adaptive thermogenesis [41], supported by alterations in plasma insulin and triglyceride levels in our findings. On the other hand, these findings provide that the elevated levels of apoptotic and pro-inflammatory markers accompanied with low-grade inflammation in PCOS animals were improved by administration of sodium butyrate, confirming the anti-inflammatory and anti-apoptotic effects of butyrate, especially in PCOS animal model.

Since GABAergic signals play a key role in orchestrating the assembly of neuronal circuits in the developing hypothalamus, and are the major inhibitory transmitters in the adult hypothalamus, it is not surprising that the dysregulation of GABAergic signaling has been associated with many neurological and neurodevelopmental disorders, such as epilepsy, schizophrenia, Down’s Syndrome and autism spectrum disorder [42]. Our data revealed a significant lowered level of GABA in the hypothalamus, this shows disruption of GABAergic signaling, which is fundamentally involved in the development of hypothalamic inflammation [42], corroborated by increased plasma SDF-1 level in this study. A study by Cassoni et al., reported that GABA and SDF-1 act synergistically, therefore resulting in tight control of cellular speed and improved directionality along migratory pathway of GnRH neurons, thus enhancing neuroendocrine [43]. Therefore, suppression of hypothalamic level of GABA contributes to hypothalamic inflammation with a decline in hypothalamic function that results in low level of GnRH, favouring androgen production in PCOS animals. Nevertheless, administration of sodium butyrate restored the level of GABA in LTZ + SOB group compared with untreated LTZ group. This observation is similar to previous studies which documented that addition of butyrate promotes GABA production [44] and decreasing pro-inflammatory cytokine secretions [45]. Overall, the present findings suggest that the short chain fatty acid, butyrate, alleviates hypothalamic inflammation associated with PCOS by improving GABA system.

## Conclusion

The present study suggests that butyrate alleviates hypothalamic inflammation in an experimental model of PCOS, a beneficial effect that is accompanied by enhanced GABA production.

## Data Availability

The data supporting the present study will be made available from the corresponding author on request.
